# Information sources, awareness and preventive health behaviors in a population at risk of Arsenic exposure: The role of gender and social networks

**DOI:** 10.1371/journal.pone.0186130

**Published:** 2017-10-09

**Authors:** Frédéric Mertens, Renata Távora, Eduardo Yoshio Nakano, Zuleica Carmen Castilhos

**Affiliations:** 1 Centro de Desenvolvimento Sustentável, Universidade de Brasília, Brasília, Distrito Federal, Brazil; 2 Community of Practice in Ecosystem Approaches to Health in Latin America and the Caribbean, Brasília, Distrito Federal, Brazil; 3 Department of Statistics, University of Brasilia, Brasília, Distrito Federal, Brazil; 4 Centro de Tecnologia Mineral (CETEM), Ministério da Ciência, Tecnologia e Inovação (MCTI), Rio de Janeiro, Rio de Janeiro, Brazil; University of Texas Medical Branch at Galveston, UNITED STATES

## Abstract

The population of Paracatu is at risk of Arsenic (As) exposure associated with long-term exploration of the largest open pit gold mine in Brazil. As part of the interdisciplinary research “The Paracatu project: Arsenic environmental contamination and human health risks assessment in Paracatu-MG”, carried out between 2011 and 2013, we used data disaggregated by gender to identify the sources of As-related information being accessed by inhabitants of Paracatu and to examine if access to these sources was correlated to awareness of As health effects and adoption of behaviors to reduce risk of As exposure. Semi-structured, face-to-face interviews were carried out with 460 participants (294 women and 166 men) to collect data on respondent’s socio-demographic characteristics, use of mass media and social communication networks as sources of information on As issues, the trustworthiness of these information sources, awareness of As health effects, and adoption of behaviors to reduce As exposure. For both men and women, interpersonal communication was used and trusted more frequently than mass media to obtain information on As. Discussion of As issues occurred preferentially among individuals of the same gender and was associated with awareness of As health risks. There are marked differences in variables correlated with the adoption of behaviors to reduce the risk of As exposure between men and women. Discussing As issues with women was associated with adoption of risk-reduction practices for both genders. In contrast, men who discuss As issues with other men were less likely to adopt As exposure prevention behaviors. Finally, adoption was associated with awareness of As health effects for women, but this was not the case for men. Policy implications for decision makers, practitioners and researchers are discussed, based on concrete examples of how gender-specific approaches can effectively guide the formulation and implementation of health promotion campaigns and programs.

## Introduction

The largest open pit gold mine in Brazil, the “Morro do Ouro”, is located in Paracatu, Minas Gerais State, a city of approximately 85,000 inhabitants. Industrial gold mining activities began in 1976–77 and are estimated to continue until 2032. Mining operations are expected to process an average of up to 55 million tons per annum (Mtpa) from 2014 to 2018. While the gold ore deposits present low Au grade from 0.4 to 0.6 g Au t^-1^, there are very high Arsenic (As) levels, over 4,000 ppm [1]. Gold mining activities contribute to the release of As in the environment, putting the population at potential risk of exposure [2]. A variety of adverse health effects have been attributed to As exposure, including skin and internal cancers, diabetes, other non-carcinogenic effects and cardiovascular diseases [3]. As such, the long-term and large-scale mining activities at the “Morro do Ouro” are increasingly a concern for local authorities and the city population.

In response to these environmental and health threats, “The Paracatu project: Arsenic environmental contamination and human health risks assessment in Paracatu” was initiated in 2011 by an interdisciplinary team of researchers and decision-makers. This research project was coordinated by the Center for Mineral Technology, part of the Brazilian Science, Technology and Innovation Ministry, and in collaboration with several Brazilian institutions and local governmental partners (see project website: http://www.cetem.gov.br/pdi/programas-e-projetos/em-curso/projeto-paracatu). This interdisciplinary project included a geochemical evaluation of As concentrations in environmental matrices surroundings the mining sites (atmosphere, soils, sediments, freshwater, groundwater and drinking water), as well as epidemiological assessment of human As exposure and potential health effects [4]. The project also included a research component to support the development of a municipal health communication and prevention program intended to increase awareness on As issues in Paracatu and to promote preventive behaviors that reduce risk of exposure. The main objectives of this latter component were to understand (i) the sources of information on As issues that were being accessed by the inhabitants of Paracatu, (ii) if these sources were trusted by the people using them and (iii) if the use of these information sources was correlated with awareness on As health effects and risk-reducing behaviors. Responses to health messages and beliefs usually differ between men and women, as do concerns and behaviors related to health risks [5–7]. As such, we applied a gender-specific perspective to data analysis and interpretation.

Two categories of information sources were investigated: mass media and social networks. Mass media, such as television, radio, newspapers and the Internet, have the advantage of reaching many people and can help bring health issues to the public agenda [8]. Yet, mass media health promotion campaigns are believed to be more effective at raising awareness, rather than achieving behavior change [8]. These campaigns are often thought to deliver messages to a population of independent individuals; however, these audiences are in fact made of groups of interacting individuals who are tied by one or more types of social relations. Accordingly, mass media campaigns can contribute to behavior change through direct and indirect routes [9]. Campaigns can be aimed at influencing directly decision-making processes at the individual level. For instance, messages that emphasize the benefits of healthy habits can elicit behavior change, based on cognitive or emotional individual responses. Mass media campaigns can also promote behavior change indirectly, by generating interpersonal communication about a particular health issue. Therefore, the influence of mass media campaigns on individuals is likely to be a function of how, with whom, and in what ways the messages are discussed and then interpreted within the context of people’s embeddedness within their complex social networks [8–10]. Accordingly, social relations and networks have increasingly been recognized as relevant information sources to be tapped by health promotion programs, building on the premises that individual health decisions are not made in social isolation but rather in interaction with others [11].

Following this perspective, scholars have embraced Social Network Analysis [12] as a research tool to understand the influence of social structures on health and well-being [13–14], as well as an intervention approach to accelerate health behavior change [15–16]. Studies on the role of social networks as a source of health information have frequently focused on the association between interpersonal communication processes and the adoption of preventive health behaviors. In fact, a positive association between these two variables has been found for a variety of health issues: family planning and reproductive health [17]; cardiovascular health intervention [18]; HIV/AIDS transmission [19]; mercury exposure through fish consumption [20] and school-based tobacco prevention programs [21], among others.

Furthermore, understanding how the trustworthiness of health information sources influences the adoption of behaviors also merits consideration. Trust in health information sources has been identified as a determinant of health risk perceptions and of behavior change [22,23]. For example, Lindström and Janzon [22] showed that trust in the healthcare system, but not the mass media, was significantly correlated to the probability of quitting smoking. Trust is also likely to determine the choice of health information sources, which, in turn, may influence an individual’s decisions and behaviors based upon that information [24]. Hence, health communication campaigns are likely to increase their effectiveness when they identify and take advantage of information sources that are not only widely accessed by the population, but those that are also recognized as trustworthy channels of communication [25,26]. Compared to mass media, interpersonal communication is believed to enhance the trustworthiness of information on health issues and motivate people to act accordingly to reduce health risk [8,27].

Gender is also a key variable to be taken into consideration in studies that seek to understand the role of interpersonal communication in the adoption of preventive health behavior [20,28]. Interpersonal communication patterns on health issues are usually different between men and women [29,30]. More specifically, discussions about health issues tend to occur preferentially among members of the same gender, such that two distinct diffusion pathways can coexist in the same population; this may in turn generate different levels of awareness between men and women [30]. While diffusion pathways preferentially connect people of the same gender, the relationships between men and women are also essential for the adoption of new health practices at the household level [30]. For instance, interpersonal relations between men and women was shown to be a main factor associated with the adoption of healthy fish diet behaviors to reduce mercury exposure risk [20] and of Chagas disease prevention strategies [31]. Furthermore, several reproductive health studies carried out in different cultural and geographic contexts have shown that the adoption of contraceptive methods (reviewed in [28]) and protection against sexually transmitted diseases [32,33] were positively related to husband-wife communication regarding family planning. Reproductive health interventions that target couples, as opposed to only one gender, have also been shown to be more effective [28].

Based on a sample from the population of Paracatu, this study has three interrelated objectives: (i) to characterize mass media and interpersonal sources of information on As issues, (ii) to evaluate the level of trustworthiness of these sources and (iii) to understand the relationships between using these sources to access information on As issues, the awareness of As-related health effects, and the adoption of behaviors that reduce the risk of exposure. Given that men and women, according to their different social roles, are likely to be involved in distinct diffusion pathways and health preventive behaviors regarding As issues, data and analysis were disaggregated by gender [34].

## Methods

### Ethics statement

The study was approved by the ethical review committee of the National Commission for Ethics in Research from Brazil (CONEP/CAE 04328912.0.0000.0019).

### Study population

In September 2013, semi-structured face-to-face interviews were conducted in two neighborhoods of Paracatu (Amoreira and Paracatuzinho) that are assisted by the Health Family´s Assistance Program (HFAP). To identify the sample population, the HFAP provided a list of individuals over 40 years old and that had been residing in Paracatu for at least 20 years, distributed among 12 subdivisions, 6 in each neighborhood. This strategy was used in order to match the sample selection criteria of the health study of the Paracatu project, whose main objective was to investigate the potential health effects of long term human As exposure. Participants from the list were asked to participate using door-to-door visits in each of the 12 subdivisions with the support of HFAP representatives. When an individual from the list was not available, another adult (over 18 years old) from the same household was invited to participate. As a consequence of the sample replacement strategy, 37% of the participants were less than 40 years old, while 18% had lived in Paracatu for less than 20 years. Interviews were conducted in the homes of participants by a team of 5 undergraduate students from the University of Brasília, under the supervision of the authors. Before starting the interview, researchers and HFAP representatives read a statement to inform the participants on the research objectives and on the potential outcomes of the study. Participants were also informed that they were free to participate or not in the study, to withdraw from the interview at any time and that their identity will be kept confidential. The statement also mentioned that research results will be presented in meetings to be organized at the health posts of each neighborhood by the researchers and the HFAP representatives. Then a verbal consent was obtained from the participants, so that they were able to make an informed decision as to whether or not they wish to participate. This strategy allowed us to conduct interviews with a total of 465 individuals, distributed among the 12 subdivisions (range 19–52 participants per subdivision). Five individuals were removed from the analysis because of incomplete data collection. The semi-structured interview included questions on the respondent’s socio-demographic characteristics, the sources they used to access As-related information, interpersonal communication on As issues, awareness of As health effects, and preventive behaviors to reduce As exposure.

### Independent variables

Socio-demographic characteristics used in this study were age, education, socio-economic status and having worked in mining (past or present). Education level was the number of years of formal schooling. Socio-economic status was measured as an interval variable according to the average monthly household income from employment activities, coded as 1, 2, 3 and 4 for income up to R$ 500, from R$ 500 to 1499, from R$ 1500 and 3000, and over R$ 3000 respectively (the exchange rate at the time of the study was 2.5 R$/US$). Respondents were asked whether they had received information on As issues through the following mass media sources: radio, television, newspaper and the Internet. Respondents were then asked whether they trusted the information provided by each of the aforementioned sources. Interpersonal communication was assessed by asking respondents to name the individuals with whom they usually discussed As issues. Respondents were allowed to cite as many individuals as they could remember. For each participant, the personal discussion network was defined as the set of people with whom the individual discussed As issues. The network was characterized both in terms of its size—number of discussion partners—and gender composition—whether discussion partners were men or women. Respondents were also asked whether they trusted the information on As issues provided by each individual in their personal discussion network.

### Dependent variables

Awareness of the adverse health effects of As exposure was assessed by asking the respondent the following questions: “Have you ever heard about As?”. The answers were recorded as “yes” or “no”. Those who answered “yes” were further asked: “Do you think that As is a potential health concern?”. The answers were again recorded as “yes” or “no”. Those who answered “yes” to this second question were asked to identify As-related diseases or adverse health effects. This was an open-ended question and responses were later categorized to define the awareness variable used in the analysis. Awareness was measured as a dichotomous variable, depending on whether the respondent was aware of the potentially harmful health effects of As and whether they were able to identify at least one possible adverse heath effect associated with exposure. Health effects included the following categories: skin or internal cancers, cardiovascular consequences, neurological effects, dermatological manifestations or respiratory symptoms.

Respondents who considered As as a potential health concern were also asked the following question: “Do you adopt any measures to reduce the risk of As exposure?” The phrasing of the question was adapted to the sociocultural context of the respondent in order to ensure mutual understanding between the interviewer and the respondent. This was an open-ended question, and responses were later categorized to define the groups of risk-reducing measures presented below. Adoption of preventive behavior to reduce As exposure was measured as a dichotomous variable, depending on whether the respondent stated that they had taken at least one of the following risk-reducing measures: cleaning the house or closing house doors and/or windows to lower air dust levels; avoiding drinking tap or well water; using an air mask; avoiding spending time in areas close to the mining sites.

### Statistical analysis

Descriptive analyses involved calculations of frequency distributions, means and ranges. Chi-Square statistical tests were conducted to compare the distribution across genders of socio-demographic characteristics, access of information sources on As issues, trust in information sources, awareness of As health effects, and adoption of preventive behaviors to reduce As exposure risks. Mann-Whitney non-parametric tests were conducted to compare the size and composition of personal discussion networks between men and women. We used multivariate logistic regression models [35] to assess the relationships between socio-demographic characteristics, use of mass media information sources, and size and composition of personal discussion networks on As (independent variables) and awareness of the health effects of As and adoption of preventive behaviors to reduce risk of exposure (dependent variables).

## Results

### Socio-demographic characteristics of the population, awareness and adoption

Table 1 presents the socio-demographic characteristics of the respondents. Among the study participants (n = 460), 64% were women and 36% were men. The age of respondents ranged from 18 to 88, with an average age of 46 years. More than 60% of the respondents had not studied beyond primary school level. Over 75% of the individuals live in a household with an average monthly income below R$1500 (approximately 600$US). A large percentage of men, 30%, had previously worked or were currently working in mining activities. No statistically significant differences were found for age, education and household income distributions between men and women. Present or past work in the mining sector was significantly lower for women (Chi square p < 0.001). About 50% of the participants reported being aware of the adverse health effects of As exposure, while only 10.9% declared that they had adopted preventive measures to reduce the risk of exposure to As. Although the percentage of awareness and adoption were higher for women than for men, the differences were not statistically significant.

**Table 1:** Frequency distributions (%) of respondents’ socio-demographic characteristics, awareness of health risks of As exposure and risk-reducing behavior.

	All (n = 460)		Women (n = 294)		Men (n = 166)	
	N	%	N	%	N	%
Age (years)						
18–30	100	21.7	58	19.7	42	25.3
31–40	79	17.2	51	17.3	28	16.9
41–50	108	23.5	70	23.8	38	22.9
51–60	81	17.6	58	19.7	23	13.9
61–70	55	12.0	38	12.9	17	10.2
71–80	30	6.5	16	5.4	14	8.4
81–90	7	1.5	3	1.0	4	2.4
Education (years)						
0	34	7.4	20	6.8	14	8.4
1–9 (primary school)	247	53.7	159	54.1	88	53.0
10–12 (secondary school)	144	31.3	90	30.6	54	32.5
>13 (undergraduate studies)	35	7.6	25	8.5	10	6.0
Household income (R$/month)						
<R$500	65	14.1	49	16.7	16	9.6
R$500–1499	291	63.3	188	63.9	103	62.0
R$1500–3000	75	16.3	40	13.6	35	21.1
>R$3000	29	6.3	17	5.8	12	7.2
Works/worked in mining sector***						
Yes	69	15	19	6.5	50	30.1
No	391	85	275	93.5	116	69.9
Awareness of As health risks						
Yes	229	49.8	156	53.1	73	44.0
No	231	50.2	138	46.9	93	56.0
Adoption of preventive behavior to reduce the risk of As exposure						
Yes	50	10.9	37	12.6	13	7.8
No	410	89.1	257	87.4	153	92.2

*** p < 0.001 between men and women, Chi Square test.

### Use and trustworthiness of information sources about As

Table 2 presents the frequency of use of As information sources among the study participants. Respondents use radio and television as source of information on As at a higher frequency than newspapers or the Internet. The most trusted mass media source of As information is television followed by the Internet and newspapers. Both men and women use interpersonal communication as an information source at a higher frequency than any mass communication channel. Interpersonal communication is considered to be a reliable source by over 90% of the respondents, making it the most trusted information source on As, by far surpassing mass media. No significant differences in use and trustworthiness of As information sources about were found between men and women.

**Table 2:** Frequency distribution (%) of respondents’ use of and trust in information sources on As issues.

	All (n = 460)		Women (n = 294)		Men (n = 166)	
	% of use	% of users that trust the source	% of use	% of users that trust the source	% of use	% of users that trust the source
Radio	18.0	51.8	18.0	54.5	18.1	46.7
Television	17.4	70.0	19.0	73.2	14.5	62.5
Internet	7.4	64.7	6.5	63.2	9.0	66.7
Newspapers	14.6	64.2	12.9	69.2	17.5	57.1
Interpersonal communication	28.7	91.0	29.9	89.1	26.5	95.1

### Patterns of interpersonal communication about As

For respondents who cited interpersonal communication as a source of As-related information, the size and gender composition of their personal discussion networks are presented in Table 3. Although the number of discussion partners varied greatly from one individual to the other, on average, men and women were equally as likely to discuss As issues with other people. Most discussions about As occurred significantly within same gender groups. Men and women were about two times more likely to discuss As issues with individuals of the same gender than with individuals of the opposite gender. Men and women with whom participants of either gender discuss As issues are trusted in similar proportion (around 90%).

**Table 3:** Interpersonal communication on As issues, stratified by gender, among the individuals who used interpersonal communication as a source of information on As issues.

	All (n = 132)	Women (n = 88)	Men (n = 44)
	Average number of discussion partners in personal communication networks on As issues (range)		
All individuals	4.5 (1–12)	4.7 (1–12)	4.2 (1–8)
Women	2.4 (0–12)	2.9 (0–10)	1.4 (0–6)***
Men	2.1 (0–8)	1.8 (0–7)	2.8 (0–8)**
All trusted individuals	4.1 (0–12)	4.2 (0–12)	4.0 (0–8)
Trusted women	2.2 (0–10)	2.6 (0–10)	1.3 (0–6)**
Trusted men	2.0 (0–8)	1.6 (0–7)	2.7 (0–8)***

** p < 0.01, between men and women, Mann-Whitney test.

*** p < 0.001 between men and women, Mann-Whitney test.

### Information sources and awareness

Table 4 shows the multivariate logistic regression model used to assess correlations between respondents’ socio-demographic characteristics, use of mass media information sources, size and gender composition of personal discussion networks, and awareness of adverse health effects of As exposure. Neither the respondents’ age, education level, household income nor past or present work in mining were associated with awareness. Among mass media information sources, only television was associated with awareness for both genders. Women who discuss As issues with other individuals, either men or women, were more likely to be aware of the health effects of As. For men, the likelihood of awareness was associated only with the number of discussion partners of the same gender, showing no statistically significant relationship with the number of women in their personal discussion network.

**Table 4:** Gender-specific multivariate logistic regression models showing the likelihood of awareness on As issues as a function of socio-demographic characteristics, mass media information sources and interpersonal communication on As.

	All (n = 460)		Women (n = 294)		Men (n = 166)	
	Odds ratio (95% C.I.*)	p	Odds ratio (95% C.I.)	p	Odds ratio (95% C.I.)	p
Age	1.01 (0.99–1.03)	0.39	1.00 (0.98–1.02)	0.97	1.02 (0.98–1.05)	0.27
Education	1.07 (0.99–1.16)	0.07	1.06 (0.97–1.16)	0.22	1.08 (0.92–1.26)	0.33
Household income	0.88 (0.61–1.27)	0.48	0.89 (0.57–1.38)	0.59	0.99 (0.49–2.01)	0.99
Work/worked in mining	1.65 (0.83–3.28)	0.15	2.18 (0.68–6.94)	0.19	2.05 (0.74–5.65)	0.16
Radio	2.22 (0.96–5.10)	0.06	2.01 (0.70–5.72)	0.19	2.98 (0.69–12.99)	0.15
Journal	1.09 (0.41–2.86)	0.86	0.81 (0.22–2.93)	0.75	1.63 (0.36–7.40)	0.52
Television	22.70 (6.70–76.94)	<0.001	22.36 (5.02–99.71)	<0.001	23.00 (2.51–210.72)	0.005
Internet	1.93 (0.46–8.08)	0.37	1.20 (0.18–8.04)	0.85	5.61 (0.67–47.30)	0.11
Number of women as discussion partners	1.50 (1.12–2.01)	0.007	1.44 (1.06–1.95)	0.019	1.80 (0.68–4.79)	0.24
Number of men as discussion partners	2.56 (1.54–4,26)	<0.001	2.28 (1.22–4.26)	0.010	3.26 (1.19–8.96)	0.02

* Confidence interval.

### Information sources and adoption

Table 5 presents the multivariate logistic regression model to identify associations between respondents’ socio-demographic characteristics, use of mass media information sources, size and gender composition of personal discussion network on As, and the adoption of behavior to reduce the risk of As exposure. We also added awareness as an independent variable in the model, in order to determine the potential relationships between awareness of adverse As health effects and the adoption of preventive health behaviors. There were no statistically significant relationships between socio-demographic characteristics and adoption for both women and men. Variables associated with adoption differ between women and men. Women who were aware of As health effects and who discussed As issues with other women were more likely to adopt practices to reduce the risk of As exposure. For men however, awareness was not significantly associated with adoption. Men who accessed information on As from television were more likely to adopt preventative behaviors. As for women, the likelihood of adoption for men was positively associated with the number of women as discussion partners. On the contrary, men who discuss As issues with other men were significantly less likely to adopt preventive behavior to reduce risk of As exposure.

**Table 5:** Gender-specific multivariate logistic regression models showing the likelihood of preventive behavior to reduce Arsenic exposure risk as a function of socio-demographic characteristics, mass media information sources and interpersonal communication on As.

	All (n = 460)		Women (n = 294)		Men (n = 166)	
	Odds ratio (95% C.I. *)	p	Odds ratio (95% C.I.)	p	Odds ratio (95% C.I.)	p
Age	1.01 (0.99–1.04)	0.34	1.02 (0.99–1.06)	0.17	0.98 (0.90–1.05)	0.54
Education	1.04 (0.94–1.16)	0.43	1.10 (0.98–1.25)	0.12	0.87 (0.63–1.20)	0.39
Household income	0.88 (0.53–1.45)	0.61	0.73 (0.38–1.39)	0.33	1.46 (0.49–4.38)	0.50
Work/worked in mining	1.11 (0.47–2.61)	0.81	0.20 (0.02–1.75)	0.14	2.20 (0.44–10.90)	0.34
Radio	0.84 (0.36–1.96)	0.69	0.54 (0.18–1.64)	0.28	3.42 (0.61–19.04)	0.16
Journal	1.34 (0.56–3.22)	0.51	1.15 (0.36–3.72)	0.81	1.52 (0.23–10.04)	0.67
Television	1.43 (0.67–3.03)	0.35	0.95 (0.37–2.46)	0.92	5.36 (1.02–28.10)	0.047
Internet	1.33 (0.48–3.72)	0.58	0.79 (0.19–3.28)	0.74	5.89 (0.66–52.25)	0.11
Awareness	9.59 (3.16–29.08)	p<0.001	9.67 (2.68–34.89)	p<0.001	8.06 (0.77–84.61)	0.08
Number of women as discussion partners	1.35 (1.15–1.59)	p<0.001	1.28 (1.07–1.53)	0.008	2.51 (1.16–5.40)	0.019
Number of men as discussion partners	0.84 (0.65–1.08)	0.17	1.13 (0.84–1.52)	0.43	0.24 (0.07–0.83)	0.024

* Confidence interval.

## Discussion

The primary objective of this article was to characterize mass media and interpersonal sources of information on As issues and to understand the relationships between the use of these sources, awareness on As, and preventive behavior to reduce exposure risks. The study was carried out with participants from the city of Paracatu that may be at risk of environmental As exposure due to nearby industrial gold mining activities. Because this project was carried out in response to a demand from Paracatu municipality, the research results and policy lessons and conclusions taken from this study were discussed with local authorities, which served as a basis for the formulation and implementation of a municipal As prevention health program.

Our results demonstrate the relevance and usefulness of using a gender-specific approach to analyze communication processes related to health promotion. We suggest that health communication studies would benefit from integrating a gendered perspective throughout the investigation process, starting from the definition of the research questions and objectives, to the phase of results interpretation as a basis for action. Classical methods tend to treat gender as a control variable in statistical models; yet, a research approach that collects and analyzes data disaggregated by gender, such as used in this study, is more likely to provide a deeper understanding of the complex roles that men and women play in the diffusion of new health behaviors.

There is consolidated evidence showing that health campaigns and interventions that use a gendered perspective can have positive effects on health outcomes, because men and women usually respond to health messages in diverse ways according to their specific values, norms and priorities [36,37]. Moreover, there is a growing demand for concrete examples of gender-specific approaches that can be effectively applied to health programming and policy-making [7]. Our results are of practical importance for the development of health communication campaigns that promote the diffusion of information on As issues and that seek to reduce the risk of exposure for inhabitants of the Paracatu region. They also have broader policy implications for decision-makers, practitioners and researchers involved in the formulation and implementation of health promotion campaigns and programs.

To obtain information on As issues, both men and women used interpersonal communication more frequently than mass media information sources. For a larger proportion of participants, information that was accessed through personal networks was considered to be a more trustworthy source when compared to information disseminated through mass media. Various communication studies have shown that trust is associated with perceived characteristics of relatives, co-workers or friends, such as helpfulness and concern [25,27,38]. These characteristics may contribute to enhancing the level of confidence in the messages communicated through social ties, compared to those conveyed by mass media sources. Among mass media communication channels, television was the most trusted As information source. We found that awareness of As health effects was associated with information sources that people trust more, such as television and interpersonal communication, suggesting that these communication channels have a higher potential to raise awareness. Accordingly, health information campaigns aimed at increasing awareness and promoting healthy behaviors should not only make use of communication channels that reach larger audiences, but they should also take advantage of information sources that people trust the most, even if they are accessed by a smaller proportion of the target population.

Discussions of As issues occurred preferentially among individuals of the same gender. This result is in accordance with the well-known principle of homophily, which proposes that relationships are more likely to be established and developed among individuals who are like one another, rather than between dissimilar persons [39]. A similar tendency was observed in other environmental health studies that analyzed interpersonal communication on the health effects of mercury [20] or pesticides [30] exposure. It is therefore expected that information on As issues will circulate through two distinct, although overlapping, diffusion pathways that are gender-specific. In recognizing that information tends to circulate preferentially among same-gender individuals, health information programs in Paracatu should target both men and women to avoid inadvertently concentrating information within only one gender group. Identifying what information tends to circulate through homophilous diffusion pathways should also be a priority for health information campaigns regarding other issues.

Discussing As issues with women was associated with adoption of practices to reduce exposure for both men and women. Discussion of As issues and adoption may be viewed as interdependent processes wherein women appear to play a central role. In contrast, men who discuss As issues with each other are less likely to adopt behaviors to reduce exposure risk. It is possible that men do not recognize individual preventive actions as the satisfactory solution to lower risk of As exposure, either because they consider them ineffective or because alternative strategies, such as political interventions, are preferred. Clearly, the reasons why interpersonal communication between men is negatively associated with adoption remain to be further explored through qualitative, in-depth analyses of men’s values, norms and message contents regarding As issues. Health promotion programs can build on the observation that the adoption of healthier practices is associated with information exchanges where women play an active role. Concrete approaches could include promoting communication among women, such as through women’s groups. Programs could be designed to favor discussions of health issues among men and women, for instance, by focusing interventions at the household level and promoting spousal communication.

Finally, adoption of behaviors to reduce the risk of As exposure was associated with awareness of As health effects for women, but not for men. While the relationships among awareness, attitudes, and behavior changes may be modeled in several ways [40], the classical cognitive model of diffusion of innovation considers awareness to be a predictor of behavior change [41]. For women, the links observed in this study between awareness and adoption are in accordance with the classical model. On the other hand, men tend to follow a decision-making process that is not dependent on awareness when they adopt measures to reduce As exposure risks.

The study has a number of limitations. A first limitation is the cross-sectional nature of the study, which does not allow discussion of any causal relation among variables. For instance, two non-exclusive explanations should be considered to explain the association between individual’s involvement in the communication network and awareness of As health effects. It is both possible that discussing As issues favored knowledge generation or that individuals who had higher level of awareness on the health effects of As exposure were more inclined to be involved in discussion of the subject. In either case, discussion of As issues and awareness of on As health effects may be viewed as interdependent processes. Further in-depth qualitative and ethnographic studies, which include the dimensions of gender equity and power relations, are needed to expand our understanding of the processes involved in the interactions between network patterns, awareness of As health effects, and adoption of preventive behaviors. For instance, further research should consider and analyze the content of discussions once the network partners are identified. A second limitation is that most of the participants in the study were individuals over 40 years old who had lived in Paracatu for at least 20 years. Since the study sample is likely not to be representative of the population living in the selected neighborhoods, results cannot be extended to the whole population. Therefore, we also need to be cautious in making policy recommendations, because they may not be valid for individuals who arrived more recently in the city. A third limitation is that we used an egocentric network approach, where the participants provide the information on their discussion partners. In future studies, it would be relevant to apply a complete network approach, for instance with the inhabitants of a whole neighborhood subdivision, in order to perform richer analyses on the relationships between the structural properties of communication networks, awareness and adoption. Finally, we did not distinguish relatives, co-workers, friends, health agents, etc. among network partners. We acknowledge that analyses based on a more in-depth characterization of network partners might further enrich the results of the study and allow us to gain a deeper understanding of the role of interpersonal communication and trust among the inhabitants of Paracatu.

Our results show that gender is a key variable to be considered in programs aimed at promoting the adoption of healthy behaviors through health risk awareness campaigns. Health communication campaigns often focus on informing the public, so that awareness increases quickly, and on shortening the time between awareness and adoption [8]. The results from the gender-specific analysis of awareness and adoption suggest that communication strategies aimed at raising women’s awareness on As health risks may in fact increase levels of adoption of preventive health behavior overall. However, targeting men using such strategies might be ineffective, as raising men’s awareness on As issues is not necessarily linked to adoption of healthy practices. These results indicate that health interventions should be grounded in a prior gender analysis of the local and cultural processes that govern the relationships between public awareness of health risks and adoption of preventive health behaviors. A deeper understanding of these linkages could inform the design and implementation of more effective health communication programs and campaigns that consider targeting men and women using different strategies.

## Supporting information

S1 Supporting Information Questionnaire used in the study. (DOC)

S2 Supporting Information Dataset used in the study. (XLSX)
