# Supplementary material for: Information sources, awareness and preventive health behaviors in a population at risk of Arsenic exposure: The role of gender and social networks
Source: PLoS One. 2017 Oct 9;12(10):e0186130. doi: 10.1371/journal.pone.0186130 (PMC5633188; doi:10.1371/journal.pone.0186130)
Supplement: S1 Supporting Information — (DOC) [file pone.0186130.s001.doc]

| **Projeto: Avaliação da Contaminação Ambiental por Arsênio e Estudo Epidemiológico da Exposição Ambiental Associadas em Populações Humanas de Paracatu-MG**  **QUESTIONÁRIO DE REDES SOCIAIS**  **ENTREVISTADOR:** | | |
| --- | --- | --- |
| REGISTRO PARACATU Nº | | DATA DE COLETA:  **/ /** |
| **IDENTIFICAÇÃO DO PARTICIPANTE** | | |
| NOME:  SEXO: I.** M** II.**** **F** | | |
| DATA DE NASCIMENTO: ______**/______/______ IDADE (ANOS):** | | |
| **DADOS SÓCIO-ECONÔMICOS** | | |
| ENDEREÇO: | | |
| BAIRRO: | TELEFONE: | |
| ESCOLARIDADE (até que série escolar estudou?) | | |
| OCUPAÇÃO ATUAL (há quanto tempo?) | OCUPAÇÃO ANTERIOR (por quanto tempo?) | |
| RENDA FAMILIAR:  I.****até 500 reais II.****de 500 a 1500 reais III.****de 1500 a 3000 reais IV. ****acima de 3000 reais | | |
| Já trabalhou em alguma mineração? (por quanto tempo?) | | |
| Há quanto tempo você mora em Paracatu? | | |

| **CONHECIMENTOS SOBRE O ARSÊNIO** |
| --- |
| 1. Você já ouviu falar do projeto Avaliação da Contaminação Ambiental por Arsênio e Estudo Epidemiológico da Exposição Ambiental Associada em Populações Humanas de Paracatu-MG?  0. ****Não 1. ****Sim. Explique com suas palavras o que já ouviu falar do projeto:  2. Você já ouviu falar ou sabe algo sobre o Arsênio?  0. ****Não 1. ****Sim. O que já ouviu falar ou sabe?  3. Você acha que é um problema para a saúde?  0. ****Não sabe 1. ****Não é um problema de saúde  2. ****Sim, é um problema de saúde. Explique porque você considera o arsênio um problema de saúde:  4. Que tipo de doença o arsênio pode causar?  5. De onde você acha que vem o arsênio (no ambiente)/ quais são as fontes de exposição?  6. De qual maneira você acha que o arsênio entra no corpo das pessoas?  7. Você acha que podemos medir o arsênio no corpo das pessoas?  0. ****Não sabe 1. ****Não, não é possível  2. **** Sim, é possível. Explique como:  8. O que você acha que pode ser feito para solucionar a questão do Arsênio em Paracatu?  9. Você faz alguma coisa pra EVITAR / PREVENIR / PROTEGER desse problema?  1. ****Não.  2. **** Sim. Explique o que você mudou nos seus hábitos:  10. Outros comentários que a pessoa fez ou queira fazer. |

| **FONTES DE INFORMAÇÕES SOBRE O ARSÊNIO REFERIDAS PELA POPULAÇÃO** |
| --- |
| De quais meios de comunicação você já ouviu falar sobre o arsênio?  1. **** RÁDIO. Qual informação você lembra ter recebido?  Fonte de informação confiável? **** sim **** não  (em relação a informação que a pessoa declarou ter obtido da fonte)  2. **** JORNAL ESCRITO. Qual informação você lembra ter recebido?  Fonte de informação confiável? **** sim **** não  (em relação a informação que a pessoa declarou ter obtido da fonte)    3. **** TV. Qual informação você lembra ter recebido?  Fonte de informação confiável? **** sim **** não  (em relação a informação que a pessoa declarou ter obtido da fonte)  4. **** INTERNET. Qual informação você lembra ter recebido?  Fonte de informação confiável? **** sim **** não  (em relação a informação que a pessoa declarou ter obtido da fonte)  5. **** OUTROS. Qual informação você lembra ter recebido?  Fonte de informação confiável? **** sim **** não  (em relação a informação que a pessoa declarou ter obtido da fonte) |

VOCE COSTUMA CONVERSAR COM ALGUÉM SOBRE O ARSENIO? **** SIM **** NÃO

Se afirmativo, pedir os nomes das pessoas com quem conversa ou já conversou. SOMENTE DEPOIS atribuir as informações de cada pessoa relacionada.

| Nome | Sexo | Relação (parente, amigo, colega, agente de saúde, etc.) | Trouxe informação nova sobre o arsênio? Qual? | Acredita na informação repassada por essa pessoa? |
| --- | --- | --- | --- | --- |
| 1. |  |  |  |  |
| 2. |  |  |  |  |
| 3. |  |  |  |  |
| 4. |  |  |  |  |
| 5. |  |  |  |  |
| 6. |  |  |  |  |
| 7. |  |  |  |  |
| 8. |  |  |  |  |
| 9. |  |  |  |  |
| 10. |  |  |  |  |
| 11. |  |  |  |  |
| 12. |  |  |  |  |

| NOME |  |  |  |  |  |  |  |  |  |  |  |  |
| --- | --- | --- | --- | --- | --- | --- | --- | --- | --- | --- | --- | --- |
|  | ----- |  |  |  |  |  |  |  |  |  |  |  |
|  | ----- | ----- |  |  |  |  |  |  |  |  |  |  |
|  | ----- | ----- | ----- |  |  |  |  |  |  |  |  |  |
|  | ----- | ----- | ----- | ----- |  |  |  |  |  |  |  |  |
|  | ----- | ----- | ----- | ----- | ----- |  |  |  |  |  |  |  |
|  | ----- | ----- | ----- | ----- | ----- | ----- |  |  |  |  |  |  |
|  | ----- | ----- | ----- | ----- | ----- | ----- | ----- |  |  |  |  |  |
|  | ----- | ----- | ----- | ----- | ----- | ----- | ----- | ----- |  |  |  |  |
|  | ----- | ----- | ----- | ----- | ----- | ----- | ----- | ----- | ----- |  |  |  |
|  | ----- | ----- | ----- | ----- | ----- | ----- | ----- | ----- | ----- | ----- |  |  |
|  | ----- | ----- | ----- | ----- | ----- | ----- | ----- | ----- | ----- | ----- | ----- |  |
|  | ----- | ----- | ----- | ----- | ----- | ----- | ----- | ----- | ----- | ----- | ----- | ----- |
